# Retraction: Atomic resolution studies detect new biologic evidences on the *Turin Shroud*

**DOI:** 10.1371/journal.pone.0201272

**Published:** 2018-07-19

**Authors:** The PLOS ONE Editors

Concerns have been raised that the data presented in this article [1] are not sufficient to support the conclusions drawn; the provenance, integrity and availability of the material used for the study have also been questioned. In light of these issues, the *PLOS ONE* Editors reevaluated the published article in consultation with members of our Editorial Board.

Based on our internal assessment and advice received from the Editorial Board members, the *PLOS ONE* Editors are concerned that there are not sufficient controls to support conclusions referring to human blood or physical trauma. For example, period ink and animal blood controls were not included in diffraction and STEM analyses, as would be needed to rule out alternate interpretations regarding the material on the fiber, and the creatinine findings do not provide definitive evidence of trauma or violence. Thus, we consider that the main conclusions of the article, including the following statements, are not sufficiently supported:

- “On the basis of the experimental evidences of our atomic resolution TEM studies, the man wrapped in the TS suffered a strong polytrauma”
- “the fiber was soaked with a blood serum typical of a human organism that suffered a strong trauma”
- “at the nanoscale it is encoded a scenario of great suffering recorded on the nanoparticles attached to the linen fibers”

In addition, the results of this article were based on analysis of a single fiber (approximately 1mm in length and 15μm in diameter) from the Turin Shroud. The reliance on a single small fiber taken from the Turin Shroud in 1978 calls into question the validity of statements in the Results and Conclusions sections which compare the new findings to those reported in previous studies of the Turin Shroud. It has not been demonstrated that findings from the fiber used in the *PLOS ONE* article can be generalized as applying to other samples taken from the Turin Shroud, or that contamination of the sample can be ruled out.

Furthermore, the Competing Interests statement for this article [1] should have declared that the sample was provided by the Shroud of Turin Education and Research Association Inc. (STERA).

In light of these issues, the *PLOS ONE* Editors are concerned about the validity of the conclusions and the reproducibility of the results, and so we retract this article.

EC, LDC, CG, and GF do not agree with the retraction and stand by the results in the article.
